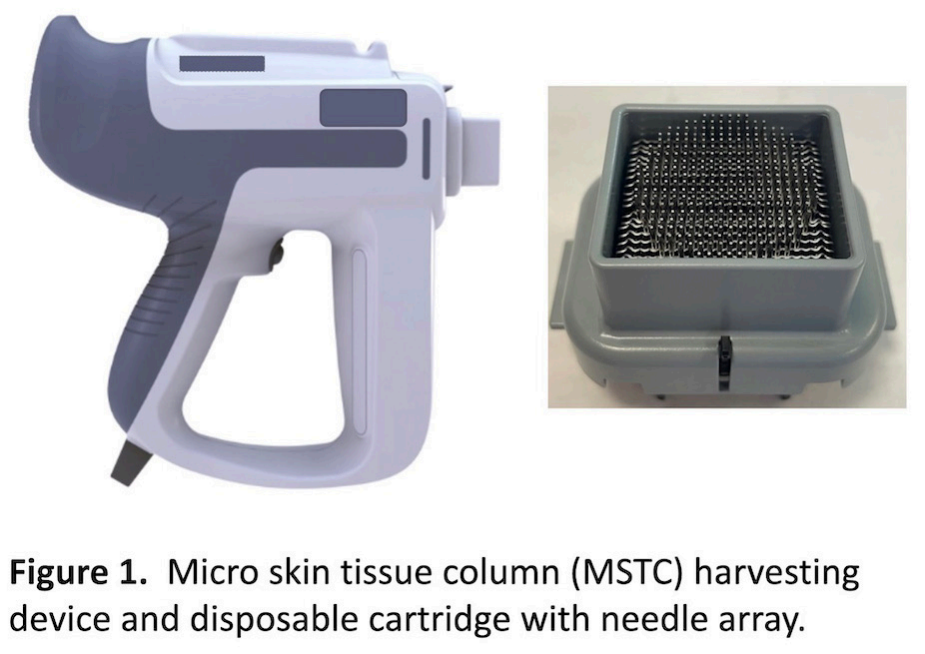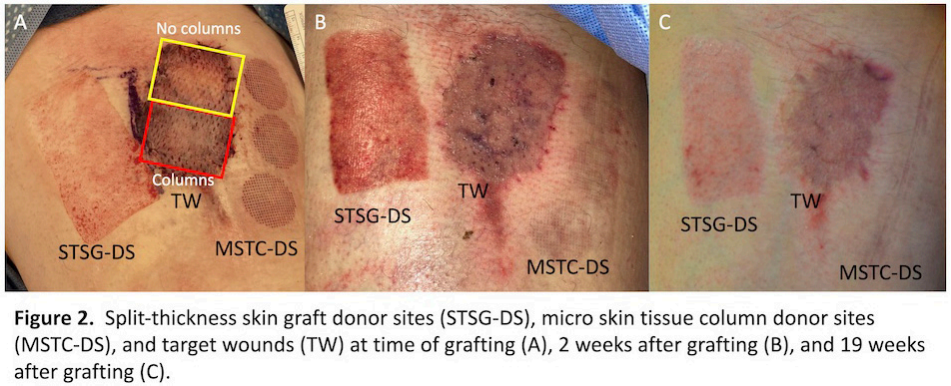# 520 A Pilot Study of Microcolumn Skin Grafting in Full-thickness Burns

**DOI:** 10.1093/jbcr/iraf019.149

**Published:** 2025-04-01

**Authors:** Martin Buta, Matthew Supple, Sean Hickey, Jonathan Friedstat, John Schulz, Jeremy Goverman

**Affiliations:** Massachusetts General Hospital; Massachusetts General Hospital; Massachusetts General Hospital; Massachusetts General Hospital; Massachusetts General Hospital; Massachusetts General Hospital

## Abstract

**Introduction:**

Grafting with micro skin tissue columns (MSTC) was developed as an alternative to split-thickness skin grafting (STSG) and full-thickness skin grafting (FTSG) for acute and chronic skin wounds. Autologous full-thickness skin microcolumns are harvested and dispersed as grafts on the target wound and have been shown to reconstitute wound volume by recapitulating normal skin components, including epidermal and dermal architecture and adnexal structures. This pilot study evaluated feasibility of intra-operative treatment of full-thickness burns with both STSGs and MSTCs. Donor sites for both were also assessed.

**Methods:**

Patients aged ≥18 years with ≤60% total body surface area (TBSA) third-degree, full-thickness burns were enrolled. One 2.5 x 2.5 cm2 wound area was treated in each subject, with the remaining portion of the wound used as an internal control. The target wound was treated with MSTCs + STSG while the control site was treated with STSG. Patients were followed for up to nine months after wound closure. Primary endpoints included re-epithelialization rate (RER), scarring (Vancouver Scar Scale, Patient and Observer Scar Assessment Scale), and pain (visual analogue scale) at graft and donor sites.

**Results:**

Ten patients were enrolled. Overall, MSTC donor sites were less painful, epithelialized faster, and resulted in improved POSAS and VSS scores than STSG donor sites. For all endpoints, there were no differences in the target wounds treated with or without MSTCs.

**Conclusions:**

Intraoperative MSTC grafting is feasible and results in minimal donor site morbidity. This pilot study was unable to demonstrate enhanced wound healing or improved scar formation when MSTCs were applied simultaneously with STSGs to burn wounds. Larger clinical studies are needed to assess the utility of MSTCs in conjunction with STSGs.

**Applicability of Research to Practice:**

This study helps clinicians understand the potential benefits and shortcomings of MSTCs in treating third-degree full-thickness burns.

**Funding for the Study:**

Funding for this pilot study was provided by Medline Industries, LP (Northfield, Illinois).